# Supplementary material for: Light-Responsive Hexagonal Assemblies of Triangular Azo Dyes
Source: Molecules. 2022 Jul 8;27(14):4380. doi: 10.3390/molecules27144380 (PMC9317042; doi:10.3390/molecules27144380)
Supplement: Supplementary file 1 [file molecules-27-04380-s001.zip › molecules-1799891-supplementary.pdf]

Supplementary Materials (SM)

Light-Responsive Hexagonal Assemblies of Triangular Azo Dyes

Mina Han and Khin Moe

Contents

Figure S1. Molecular switching of compound **1** between C_3-symmetric and asymmetric structures based on [A] ^{1}H NMR and [B] absorption spectral changes.

Figure S2. SEM image of **3** fibrous structures obtained from 40 μM toluene-MeOH (1:3, v/v) mixture.

Figure S3. (a) SEM and (b) FOM images of hexagonal rods obtained from 30 μM THF-H_2O (1/4, v/v) mixture.

Figure S4. SEM images of self-assembled structures of **1** formed in various mixed solutions.

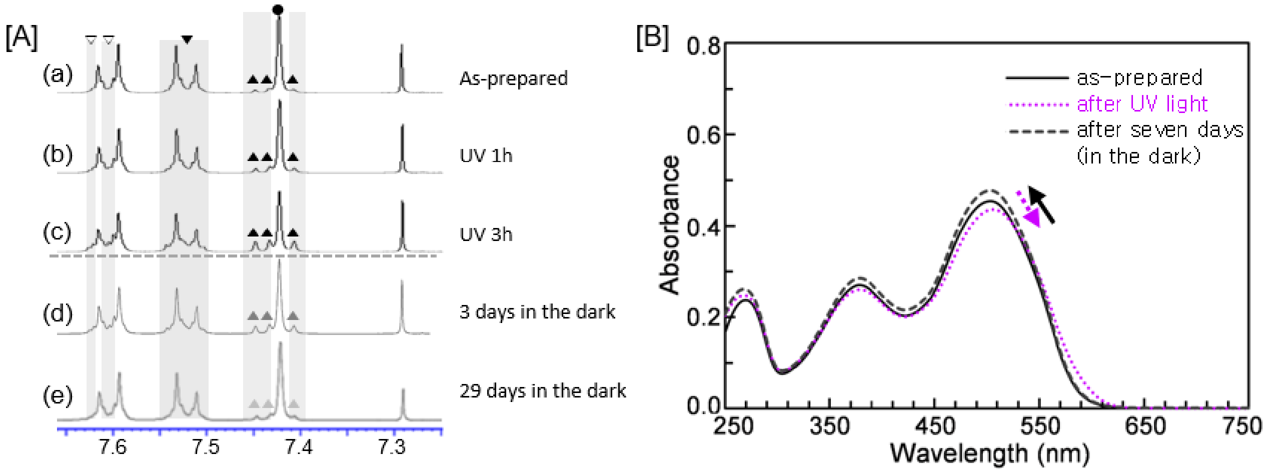

Figure S1. Molecular switching of compound **1** between C_3-symmetric and asymmetric structures based on (A) ^{1}H NMR and (B) absorption (in THF) spectral changes by UV light and heat (in the dark). (A-a) As-prepared, after exposure to UV light for (A-b) 1 and (A-c) 3 hours (C_3-symmetric/asymmetric = 65/35) and after dark incubation for (A-e) 29 days (C_3-symmetric/asymmetric = 95/5). UV light intensity: 1.5-2.0 mW/cm^2.

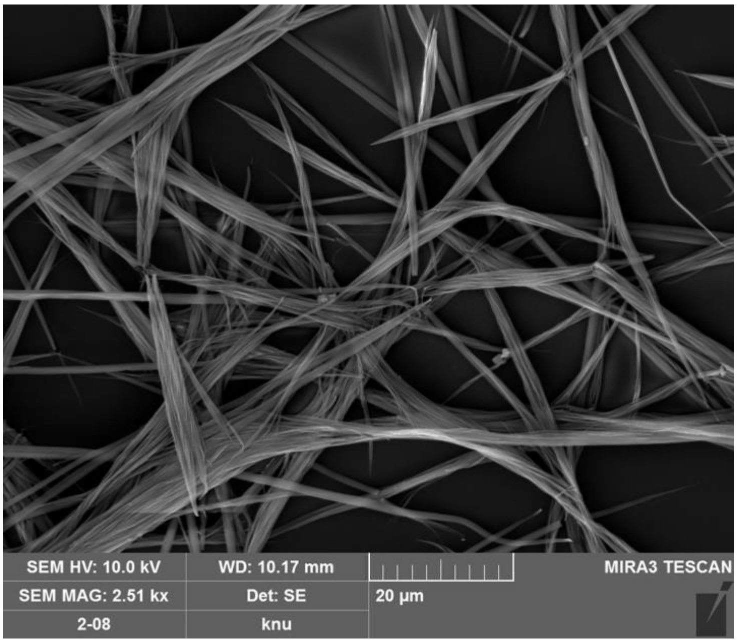

Figure S2. SEM image of **3** fibrous structures obtained from a 40 µM toluene-MeOH (1:3, v/v) mixture.

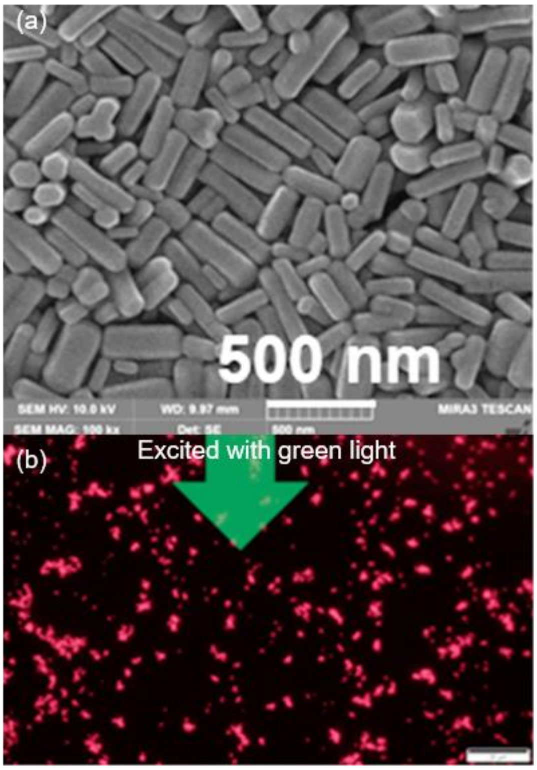

Figure S3. (a) SEM and (b) FOM images of hexagonal rods obtained from 30 μM THF-H_2O (1/4, v/v) mixture.

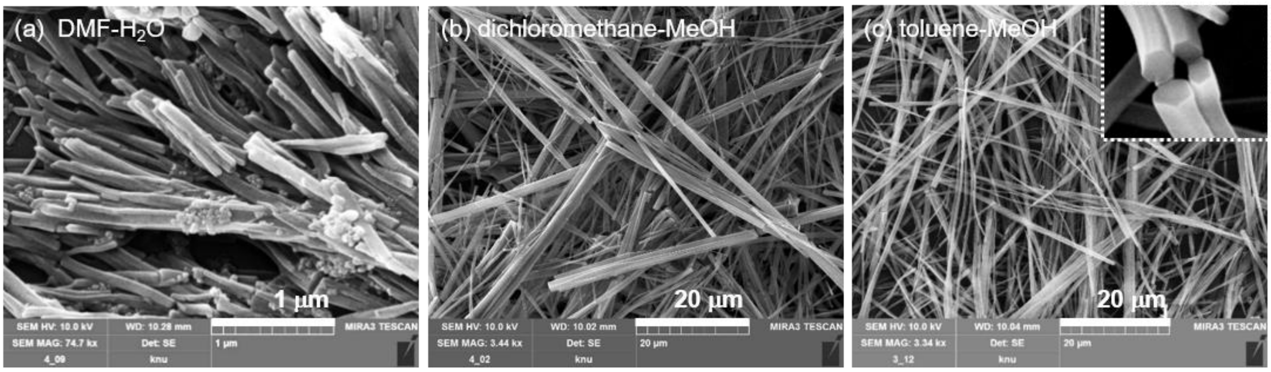

Figure S4. SEM images of self-assembled structures of **1** formed in various mixed solutions.
